# Bearded Reedlings Adjust Their Pair-Bond Behaviour in Relation to the Sex and Attractiveness of Unpaired Conspecifics

**DOI:** 10.1371/journal.pone.0032806

**Published:** 2012-02-29

**Authors:** Herbert Hoi, Matteo Griggio

**Affiliations:** Department of Integrative Biology and Evolution, Konrad Lorenz Institute of Ethology, University of Veterinary Medicine of Vienna, Vienna, Austria; University of Sussex, United Kingdom

## Abstract

An individual's investment in mating or keeping a pair bond intact may be influenced not only by the attractiveness of its current mate, but also by that of other potential mates. In this study, we investigated the effect of relative attractiveness on pair-bond behaviour in bearded reedlings, *Panurus biarmicus*. We showed that mate attractiveness, in terms of beard length in males and tail length in females, influenced courtship behaviour when the pair was kept isolated. In the presence of a conspecific, contact initiations within a pair increased. This increment was mainly related to the sex of the unpaired conspecific, however, and less to differences in attractiveness between the current partner and the unpaired conspecific. Female contact initiations towards potential extra mates were independent of male attractiveness, whereas male contact behaviour was significantly influenced by female attractiveness. However, females displayed more contact initiations to their current mate when they were less attractive than the unpaired females. Males decreased their overtures towards other females with increasing attractiveness of their current mates. Overall, our results suggested that, when there was a risk of losing their mate, bearded reedlings adjust their pair-bond investment mainly in response to the presence or absence of a competitor, and fine-tune investment to a lesser extent in response to the attractiveness of that potential competitor.

## Introduction

Animal decisions and their actions often occur in public [1]–[6]. As a result, individuals can use social information to make decisions, and they may adjust their behaviour whenever conspecifics are present [7]–[11]. The question of how the presence and relative attractiveness of an unpaired conspecific affects the behaviour of pair-bonded individuals has, however, received little attention [12]–[14]. In particular, an individual's investment in mating with a conspecific or maintaining the pair bond may be influenced not only by the attractiveness of its current mate, but also by that of other potential mates. Whenever mate choice is constraint, post-mating sexual selection may significantly influence the social relationship between pair mates, particularly in birds, where only a small fraction of socially monogamous species show monogamy on the genetic level, and the intensity of sexual conflict can be intense [15]–[18]. As male and female interests frequently differ with regard to fertilisation success [19]–[23] the mating interactions between males and females are not necessarily cooperative. In fact, sexual reproduction is burdened with conflict, and the potential for mates to exploit one another may even lead to divergence in sex roles (reviewed in [24]). Petrie & Kempenaers [25] pointed out that monogamous females are restricted in their choice of partners, since only one female can be paired with the best male. When paired with a low-quality male, females should try to seek extra-pair copulations [17], [25]–[27] or divorce when a better option arises [28], [29]. A quality mismatch between pair members, however, may also be reflected in the investment in pair-bond behaviour [27], [30]. Petrie & Hunter [31] and Petrie & Kempenaers [25] predicted that, in socially monogamous species, the relative quality of the members of a pair is an important determinant for who invests more in maintaining the pair bond, especially when another option appears (i.e. a potential extra-pair or social mate). Hence, the degree of conflict depends on the attractiveness of the two pair members and another potential social or copulation partner [30], [32]. This degree of conflict may then determine the individual behaviour towards other available individuals (e.g. investment in extra-pair behaviour, defence behaviour) or towards the current partner (e.g. mate guarding or other behaviours to maintain the pair bond) [32]–[35]. A cost of this sexual conflict related to individual variation in attractiveness is either the lost of paternity or the lost of the mate.

In bearded reedlings (*Panurus biarmicus*), by settlings in colonies, attractive females increase their opportunities to incite male-male competition for extra-pair copulations and, consequently, to secure extra-pair fertilisations by attractive males [36]. Although pair formation occurs prior to the first reproductive period and a pair usually remains together for life [37], extra-pair paternity in colonial breeding pairs is very high, suggesting post-mating sexual conflict. Female bearded reedlings select their mates according to morphological characters like beard length [38] and tail length [39]. Hoi & Hoi-Leitner [36] showed that beard length also affects paternity uncertainty, indeed, males with longer beards had fewer extra-pair chicks in their brood than short-bearded individuals. There is evidence that males prefer females with longer tails [39], probably because female tail length is an indicator of female fecundity [36]. Using these morphological measurements as indicators of attractiveness, we examined how courtship behaviour, i.e. direct contact initiation (non-disturbing approaches performed by the paired individual) towards the opposite sex and aggressive behaviour among members of the same sex was influenced by the attractiveness of paired individuals in relation to unpaired conspecifics and competitors.

In our study we presented a focal pair (focal male plus a focal female) with an unpaired conspecific who was either male or female. With this experimental approach on bearded reedlings we were able to explore whether a same-sex and a different-sex conspecific would affect partner pair-bond behaviour. At the same time, we were able to simulate the situation of individuals being faced with conflicts in such a triangle situation [24], and to test how investment in behaviours related to maintaining a pair bond, or building up a relationship with another conspecific, are driven by attractiveness. We predicted that bearded reedlings should adjust their pair-bond investment in response to the presence or absence of a competitor, and should adjust their investment in response to the attractiveness of that potential competitor.

## Methods

### Ethics statements

Prior to conducting the experiment, we had decided to suspend the experimental trial should the unpaired bird or the focal bird became hurt or critically distressed, but at no time did we have to intervene. None of the unpaired or focal birds died during the experiment. Immediately after the experiment, birds carried out several successive breeding attempts, suggesting that the housing conditions were appropriate and that the experimental birds remained healthy. Licenses to take and keep birds from the field were given by the Burgenländische Landesregierung (No. IV-1253/38; IV-1058/39; and 5-N-A1007/178 based on the ‘Burgenländisches Naturschutzgesetz’: LGBI.Nr. 22/1980). The experiments reported in this paper comply with the current laws on animal experimentation in Austria and the European Union.

### Study species and experimental design

The socially monogamous bearded reedling almost exclusively inhabits extended areas of reed beds [37]. Although male and female remain together for life, females regularly initiate extra-pair copulations by soliciting copulations from other males [36]. When conflicts related to this extra-pair behaviour arise, pairs have usually already settled and the costs of divorce may be high in comparison with benefits maintaining a pair-bond (e.g., increased synchronisation resulting in earlier start of breeding [35], [40]). In this study, we experimentally delayed pair formation, allowing birds to pair just two weeks prior to the start of breeding. This enabled us to create a situation in which pairs with pair bonds that were still weak (low divorce costs) could encounter other conspecifics as potential social or copulation partners.

Bearded reedlings were captured in autumn in the reed beds of Lake Neusiedl (Eastern Austria) and housed in outdoor aviaries measuring 7×5×3 m at the Konrad Lorenz Institute of Ethology, Vienna (KLIVV). Here, we carried out the study in December and February, some weeks prior to the start of breeding. The housing aviaries were equipped with reeds and water basins to replicate the birds' natural environment, and food (commercial food for insectivorous passerines, mealworms, ant pupae, crickets and a variety of seed types) was provided ad libitum. Birds were kept in single-sex flocks and birds used in the experiment had no prior contact with each other.

### Experimental set-up

Each experimental pair (focal pair) was formed from a randomly chosen male and a randomly chosen female. The focal pair was introduced into an outdoor aviary (5×3×2 m) and visually and vocally isolated from other birds for one week. Since pair formation usually takes only a few days (usually one week, authors' personal observations), and there were no other birds to choose from, the individuals had no alternative but to pair with the available mate. After one week, an additional bird (unpaired bird) was introduced into the aviary for a period of four hours (from 12 h to 16 h), and every second day a new bird was introduced in the same way. A total of fourteen focal pairs were tested in this way, with two unpaired males and two unpaired females respectively. In total, five unpaired males and five unpaired females were introduced to these fourteen focal pairs. When not being used in the experiment, unpaired birds were kept isolated in cages (100×50×50 cm) and given at least six days to recover between experiments.

We measured courtship behaviour (within the pair and towards the extra bird) and agonistic behaviour (towards the unpaired bird) of the focal birds in 30-minute observation periods. Agonistic behaviour was defined as an aggressive chase-flight performed towards the unpaired bird. Chased birds always flew away and never started a fight. Direct contact initiations were defined as non-disturbing approaches performed by the paired individual towards an unpaired bird of the opposite sex (i.e., when a focal bird followed within 2–5 sec after the departure of the unpaired bird, or approached usually with a physical contact the unpaired bird from a distance of at least 2 m).

Two behavioural observations were performed for each focal pair when: (i) the pair was kept isolated (one week after being released into the aviary; first behavioural observation at 12 h and second session three hours later); (ii) the pair was with an unpaired male; and (iii) the pair was with an unpaired female. When an unpaired bird was presented to the pair, the first behavioural observation was undertaken just after the bird was released into the aviary with the pair, and the second behavioural observation three hours later.

### Scoring attractiveness and mismatch

We scored focal-male attractiveness in terms of the mean beard length [38] of the fourteen focal males, and focal-female attractiveness in terms of the mean tail length of the fourteen focal females. Deviation from mean values was then used as an index of attractiveness.

To calculate the degree of mismatch in attractiveness within pairs, we ranked each of the fourteen focal females and fourteen focal males from 1 (most attractive) to 14 (least attractive). The degree of mismatch within a focal pair was then derived from the difference between attractiveness ranks. In ten pairs the female was more attractive (had a lower rank value) than her mate, whereas in four pairs the male was more attractive (lower rank value) than his mate.

In order to establish whether a focal pair was tested with a more or less attractive unpaired bird, we used the absolute differences in beard length (males) and tail length (females) between focal-pair members and unpaired-pair birds as an index of their relative attractiveness.

### Statistical analyses

For isolated pairs, multiple regression analyses were performed for each sex to examine the consequences of mate attractiveness and pair mismatch on the number of direct contact initiations, which was normally distributed after log-transformations. We expected the response of the focal birds to be stronger towards the unpaired bird with an increase in relative attractiveness between focal and unpaired birds. Therefore we tested two unpaired males and two unpaired females with each pair, but in the analyses we included only data from the unpaired bird which differed most in attractiveness from the focal bird (i.e. for each pair, one unpaired male and one unpaired female experiment). Using this criterion, five focal pairs were confronted with an unpaired male more attractive than the pair male, and nine with a less attractive male. Four unpaired males entered the analyses three times and one male twice. In the unpaired female experiments, seven focal pairs were confronted with a more attractive unpaired female and the other seven with a less attractive female. Again four unpaired females entered the analyses three times and one female twice. We used repeatability analyses [41], [42] to evaluate whether the behavioural response of females is repeatable in the presence of a specific unpaired male or whether male response is repeatable in the presence of a specific unpaired female. We found no repeatability in terms of female contact initiations/h towards the male partner (p>0.4) or the unpaired male present (for both, p>0.3). The same is true for male contact initiation/h towards the female pair partner (p>0.6) or an unpaired female (p>0.5). This suggests no major individual effect of the extra birds used and therefore reduces the likelihood for a strong effect of pseudoreplication. To examine whether pair behaviour is effected by sex and attractiveness of the unpaired bird housed together with them we used the proportion of female courtship behaviour in relation to the total number of contact initiations by both sexes as dependent variable in a Generalized linear model with a 2×2 factorial design. The sex of the unpaired bird (male or female) was crossed with its relative attractiveness (more attractive or less attractive). Male and female extra male entered as covariate. An angular transformation (arcsin square-root) was used to achieve a normal distribution of the proportional data. Spearman rank correlations were used to investigate the effect of unpaired bird attractiveness on direct contact initiation and aggressive behaviour within focal pairs, and towards unpaired birds. Finally, Bonferroni adjustments were shown for statistically significant results.

## Results

### Direct contact initiation within the pair without unpaired birds

On average, both sexes invested almost equally in keeping contact. Focal females initiated about 58% of all contacts (mean (± SE) number of contacts initiated/h: females: 8.00±1.13, males: 5.8±1.19, p = 0.09, t = −1.7, N = 14; paired t-test).When we controlled for the degree of mismatch within pairs, however, a multiple regression analysis showed that individual contact initiations were related to mate attractiveness. In focal males, courtship behaviour significantly decreased with increasing attractiveness of their mates (multiple regression model: F = 11.7, df = 2,14, r_part_ = −0.72, p = 0.01). The pair mismatch, however, did not influence courtship behaviour when we controlled for attractiveness (r_part_ = 0.023, p>0.8). Female contact initiations, on the other hand, were positively correlated with male attractiveness (F = 9.8, df = 2, 14, r_part_ = 0.56, p = 0.049), but not with pair mismatch (r_part_ = 0.01, p>0.9).

### Direct contact initiation within the pair with unpaired birds

**Figure 1:**
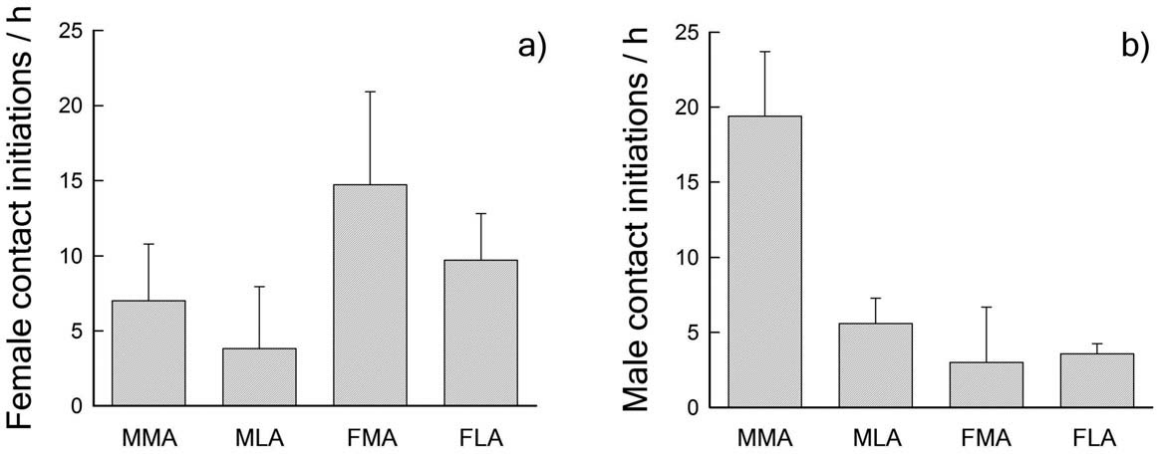
Frequency of contact initiations between pair members in the presence of an unpaired bird in comparison with being kept isolated (0 - line) and in presence of an unpaired bird. MMA/MLA: unpaired male more/less attractive than focal male; FMA/FLA: unpaired female more/less attractive than focal female. Given are means (± SE) of (a) females and (b) males.

**Figure 2:**
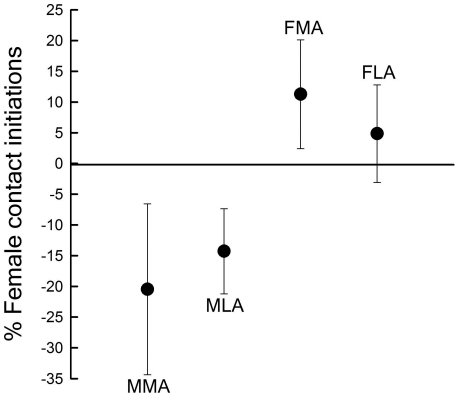
Proportions of female contact initiations towards her partner (being kept isolated marked by 0 - line) or in relation to the total number of contact initiations by both sexes in the presence of an unpaired bird. Negative deviations from the 0-line denote a decrease in female contacts and, consequently, an increase in male contacts. MMA/MLA: unpaired male more/less attractive than focal male; FMA/FLA: unpaired female more/less attractive than focal female.

Focal males and focal females generally increased the number of contact initiations towards their mate when an unpaired bird was present (Figure 1). A detailed investigation revealed, however, that this increase varied according to the sex of the unpaired bird (Table 1). Females significantly increased direct contact initiations only in the presence of a potential competitor (unpaired female), but not when an unpaired male was present. Male contact initiations towards the partner increased significantly when a potential competitor (unpaired male), whether more attractive or less attractive than the focal male, was present, but also in the presence of an unpaired female less attractive than the partner (Table 1). Furthermore to examine whether pair behaviour is sensitive to sex and attractiveness of the unpaired bird housed together with them, we used the proportion of female courtship behaviour in relation to the total number of contact initiations by both sexes. In a Generalized linear model this variable revealed a significant effect of sex (F = 17.4, p<0.001, df = 1,28) and to a lesser extend of attractiveness (F = 2.8, p = 0.08, df = 1), and there was an almost significant interaction between the two (F = 4.2, p = 0.051, df = 1,28). The proportion of female courtship behaviour decreased in the presence of an unpaired conspecific, both more or less attractive than her current partner but increased in the presence of a more or less attractive unpaired female (Figure 2).

**Table 1:** Mean ± SE female and male contact initiations/h in presence of an unpaired bird that was either more or less attractive than the focal bird.

	*Female contact*	*Male contact*
*Unpaired female*		
*More attractive (7)*	24.71±6.2*	10.43±3.7
*Less attractive (7)*	15.71±3.1*	7.86±0.7*
*Unpaired male*		
*More attractive (5)*	13.60±3.9	24.00±4.3*
*Less attractive (9)*	12.67±4.2	12.11±1.68**

Asterisk denotes significant differences(1) between the number of direct contact initiations when the pair was alone and when an unpaired bird was present. The number of tested pairs is given in brackets.

(1) probability values from paired t-tests:

* p<0.05;

** p<0.01.

**Figure 3:**
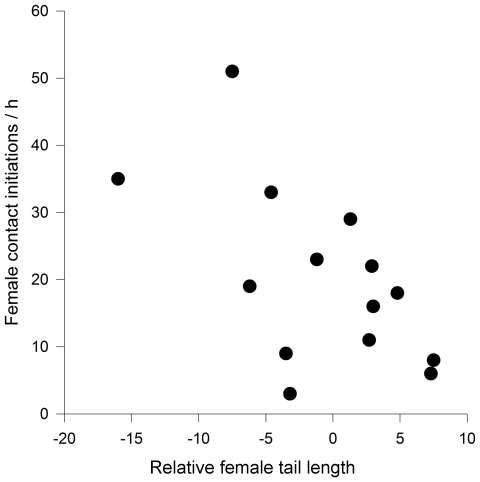
Relationship between a female's contact initiations towards her partner and her tail length relative to that of an unpaired female, which indicates her relative attractiveness. Attractiveness of the focal female increases relative to the attractiveness of the unpaired female along the x-axis.

Female relative attractiveness, however, negatively influenced the number of direct contact initiations towards the mate in the presence of an unpaired female. The more attractive a focal female was relative to the unpaired female, the less she contacted her partner (r_s_ = −0.60, p = 0.02, N = 14; Figure 3). On the other hand, we found no correlation between female relative attractiveness and male courtship behaviour in the presence of an unpaired female (r_s_ = 0.12, p>0.3, N = 14), nor between male relative attractiveness and female (r_s_ = 0.01, p>0.8, N = 14) or male (r_s_ = 0.24, p>0.2, N = 14) courtship behaviour in the presence of an unpaired male (r_s_ = 0.2, p>0.2, N = 14).

### Direct contact initiations towards other unpaired birds

**Figure 4:**
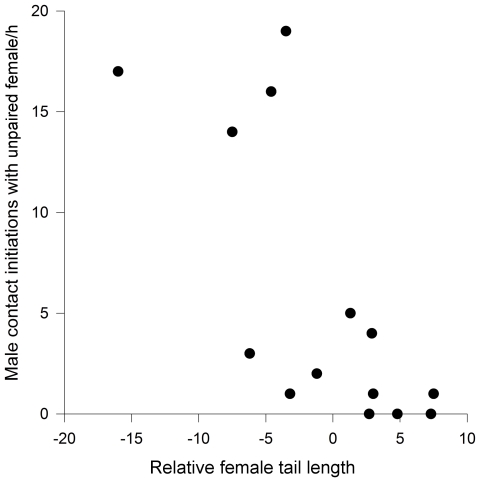
Relationship between a male's contact initiations towards an unpaired female and the relative tail length of his partner. The focal female's attractiveness increases in relation to the unpaired female's attractiveness along the x-axis.

The results demonstrated that female courtship behaviour towards unpaired males was not related to the relative attractiveness of their mates (r_s_ = −0.06, p>0.8, N = 14; Table 2). Female relative attractiveness, however, influenced male direct contact initiations towards unpaired females. On average, males most frequently contacted females of better quality than their mates (Table 2). Males decreased their courtship behaviour towards unpaired females as the relative attractiveness of their partners increased (r_s_ = −0.75, p = 0.01, N = 14; Figure 4).

**Table 2:** Mean (±SE) female and male contact initiations/h towards an unpaired conspecific. The number of tested pairs is given in brackets.

	*Unpaired partner*	
	*More attractive*	*Less attractive*
*Female contact*	5.80±2.4 (5)	2.90±1.1 (9)
*Male contact*	10.30±2.9 (7)	1.60±0.8 (7)

### Aggressive behaviour towards competitors

Focal males and focal females both behaved aggressively towards unpaired birds of the same sex (Table 3). These shows of aggression, however, did not correlate with the attractiveness of the unpaired bird relative to themselves, either in the case of female aggression towards an unpaired female (r_s_ = 0.24, p>0.4, N = 14) or in the case of male aggression towards an unpaired male (r_s_ = −0.38, p = 0.17, N = 14).

**Table 3:** Mean (± SE) female and male aggressions/h towards a potential competitor (unpaired bird) that was either more or less attractive than themselves.

	*Competitors*	
	*More attractive*	*Less attractive*
*Female aggressions*	57.43±27.5 (7)	151.28±73.2 (7)
*Male aggressions*	100.80±51.1 (5)	69.86±40.8 (9)

The number of tested pairs is given in brackets.

## Discussion

Firstly, our results showed that, in the presence of a conspecific, contact initiations within a pair increased in comparison to the situation in which the pair was isolated. This increment was, however, mainly related to the sex of the conspecific. Indeed, females significantly increased direct contact initiations only in the presence of an unpaired female, but not when an unpaired male was present. Male contact initiations towards the partner increased significantly when an unpaired male was present, but also in the presence of an unpaired female if she was less attractive than his partner. Secondly, our results showed that female contact initiations towards unpaired males were independent of her partner's attractiveness, whereas male contact behaviour was significantly influenced by female attractiveness. Males decreased their contacts towards other females with increasing attractiveness of their current mates. Overall, our results suggested that, when there was a risk of losing their mate, bearded reedlings adjusted their pair-bond investment mainly in response the presence or absence of a competitor, and fine-tuned this investment to a lesser extent in response to the attractiveness of that competitor. So, it seems that males were concerned with possible opportunities to trade up from their partner, while females were concerned with competition from rival females who might provide those trading-up opportunities. Both sexes were sensitive to the attractiveness of unpaired females, relative to the focal female.

Petrie & Hunter [31] suggest that conflict intensity might be reflected in the investment of pair partners in pair-bond behaviours (i.e. direct contact initiations). Specifically, the less attractive mate should invest more in keeping the contact. Our results demonstrated that, when the pair was kept isolated, both sexes invested equally in direct contact initiations, and we found no effect of mismatch in attractiveness between sexes in this behaviour. Partner attractiveness, however, had a clear effect (e.g. [43], [44]). Females increased their pair-bond behaviour with the attractiveness of their partners in terms of beard length. Males, surprisingly, initiated fewer contacts when their mates were more attractive (in terms of tail length). In general, one would expect that individuals would prefer the most attractive partner [45]. Gowaty [22], however, pointed out that this is not necessarily always the case, since the probability of females engaging in extra-pair matings could influence male mate choice, and therefore less attractive males may take this into account when choosing a mate. In bearded reedlings, extra-pair paternity does occur, mainly driven by females, which also decide where to nest [36], [46]. Attractive females prefer to settle in aggregated breeding situations, which increase their opportunities to adjust their choice of partner by obtaining extra-pair copulations [36]. Moreover, female bearded reedlings do not apparently suffer any disadvantage (e.g. reduced parental effort or punishment by the partner) from engaging in extra-pair behaviour [36]. Thus, less attractive males may prefer attractive females only for extra-pair copulations, but may otherwise prefer less attractive females as social partners. This is supported by a male choice experiment in relation to female tail length, the results of which suggest that males follow a double strategy: they choose a social partner with a medium tail length but at the same time display to other females with longer tails [39]. Thus, males do not necessarily follow the same rules in selecting a partner as females [47]–[49], and our experiment suggests that female faithfulness could be one important factor in mate choice [50].

Theoretical studies suggest that variation in the attractiveness of pair partners in relation to potential mates may influence the intensity of sexual conflicts [16], [22], [25], [27], [31]. In the presence of a better option, individuals are expected to decrease contact initiations towards their mate and increase them towards potential extra-pair partners. In contrast with this prediction, we found that only female relative attractiveness influenced courtship behaviour within the pair. In the presence of a competitor, a female's contact behaviour towards her partner decreased when her attractiveness relative to the unpaired female increased. Additionally, the male increased his contact initiation towards his partner in the presence of an unpaired female less attractive than the female partner. Moreover, male direct contact initiations towards an unpaired female decreased with increasing attractiveness of their mates. Male relative attractiveness, however, did not have a significant effect on either pair-bond behaviour within the pair or on female contact initiation towards potential mates. On the other hand, we found that, in general, both members of a pair contacted each other more often in the presence of an unpaired bird, and so we cannot say that contact initiation within the pair decreased when a more attractive partner was available. Furthermore the results revealed that changes in courtship behaviour were more strongly influenced by the sex of the unpaired bird than its attractiveness. Even in the case when both partners are attractive and their risk of mate loss or extra-pair paternity should therefore be low [25], [30], [31], contact initiations within the pair increased. Contact initiations within the pair can therefore be seen as a form of mate guarding and/or mate retention behaviour for both pair members [51].

With regards to aggressive behaviour towards competitors, both males and females defended their partner in the presence of a competitor. This result seems logical since, in the presence of competitors, individual reproductive fitness is at stake. Individuals already mated should therefore try to keep their partner, whereas attractiveness (in terms of secondary sexual characters) should remain of secondary importance. Since aggressive behaviour towards competitors was independent of attractiveness in both males and females, this assumption seems very likely.

A possible function of the extended pair-bond period is to promote the sharing of parental care. In bearded reedlings, parental care is provided by both male and female, and the benefits of maintaining the existing pair bond (i.e. accumulation of breeding experience, earlier laying date, improvement in reproductive success [35], [52]) outweigh the benefits involved in mate switching. It has been shown that male bearded reedlings do not withdraw their parental care when their partners engage in extra-pair copulations [36], [46]. Therefore, given the opportunity to choose between the current partner and a more attractive individual in terms of secondary sexual traits, bearded reedlings do not seem to switch mates. Pair partners rather prefer to stay with their current partner, and there is no indication of a conflict owing to different interests being reflected in their mating behaviour [25], [27], [31].

To conclude, in the presence of a conspecific, contact initiations within a pair increased, and bearded reedlings adjusted their pair-bond behaviour to the partner, when at risk of losing their mate or of losing paternity, and fine-tune investment to a lesser extent in response to the attractiveness of that potential competitor.
